# Dopamine-Assisted Modification of Polypropylene Film to Attain Hydrophilic Mineral-Rich Surfaces

**DOI:** 10.3390/polym15040902

**Published:** 2023-02-11

**Authors:** Alenka Ojstršek, Angela Chemelli, Azra Osmić, Selestina Gorgieva

**Affiliations:** 1 Institute of Engineering Materials and Design, Faculty of Mechanical Engineering, University of Maribor, Smetanova ulica 17, 2000 Maribor, Slovenia; 2 Institute of Inorganic Chemistry, Graz University of Technology, Stremayrgasse 9, 8010 Graz, Austria; 3 Institute for Automation, Faculty of Electrical Engineering and Computer Science, University of Maribor, Koroška cesta 46, 2000 Maribor, Slovenia

**Keywords:** polypropylene film, dopamine-assisted modification, hydrophilicity, mineralization

## Abstract

The presented study focuses on the modification of polypropylene (PP) film with tetraethyl orthosilicate (TEOS) under heterogeneous conditions via polydopamine/polyethylene imine (PDA/PEI) chemistry using a facile dip-coating procedure to attain hydrophilic mineral-rich surfaces. Thus, the resulting PP-based films were further immersed in ion-rich simulated body fluid (SBF) to deposit Ca-based minerals onto the film’s surfaces efficiently. In addition, the chemical reaction mechanism on PP film was proposed, and mineralisation potential inspected by determination of functional groups of deposits, zeta potential, hydrophilicity and surface morphology/topography using Fourier transform infrared (FTIR) spectroscopy, streaming potential, water contact angle (WCA), scanning electron microscopy (SEM) and atomic force microscopy (AFM). The obtained results show the improved wettability of samples on account of PDA inclusion (WCA was reduced from 103° for pure PP film to 28° for PDA-modified film), as well as the presence of functional groups, due to the PDA/PEI/TEOS surface functionalisation, increased the ability of minerals to nucleate on the PP film’s surface when it was exposed to an SBF medium. Moreover, the higher surface roughness due to the silica coatings influenced the enhanced anchoring and attachment of calcium phosphate (CaP), revealing the potential of such a facile approach to modify the chemically inert PP films, being of particular interest in different fields, including regenerative medicine.

## 1. Introduction

Biomineralization is a process that occurs in the skeletal system of healthy mammals, resulting in bone and tooth (dentin and enamel) tissues, all being composite materials containing water and inorganic and organic components [1,2]. The inorganic mineral component of bone is a nanosized, elongated platelet-like carbonated calcium phosphate (CaP), the particles of which are aligned along the type I collagen fibril, being compositionally and structurally similar to the synthetic stoichiometric hydroxyapatite (HA), Ca_5_(PO_4_)_6_(OH)_2_ [3]. Due to its poor fatigue-resistance, it needs to be integrated into an interpenetrating matrix for bone-related therapies, enhancing the strength and toughness of CaP minerals [4]. CaP–based materials also have proven regeneration effects in applications such as implants interfacing with bone tissue (e.g., biomaterials for articular cartilage repair). In this context, the CaP-rich, mineralised polymeric surfaces used as regenerative barriers are also highly relevant in bone regeneration procedures. The fundamental characteristics of regenerative barriers are associated with properties such as biocompatibility, cell exclusion, clinical handling, and integration into host tissues [5]. Generally, the efficiency of the material in this area is related to the synergistic effect of structuring, composition, porosity, presence of active agents, etc., and characterisation set-ups normally combine the effects. Moreover, microscale and nanoscale roughness can affect biomineralization directly in a way that various structural features of the materials regulate the proliferation or differentiation potential of the cells [6]. 

The types of polymeric materials available to in-bone regeneration research are inexhaustible. The impermeable, non-resorbable polypropylene (PP) barrier with high rigidity and elasticity recently demonstrated feasibility for use in the (modified) guided bone regeneration (GBR) technique, implying its easy insertion in blood clots without the need for grafting, and, consecutively, easy removal after surgery [5,7,8]. Resende et al. [9] reported that when the PP membrane was exposed to the oral environment it minimised infiltration of adjacent tissue cells, favouring the proliferation of bone cells within the alveolus without the risk of bacterial contamination. Moreover, PP manifests excellent thermal and chemical stability, light weight, well-controlled porosity, easy processing, low cost, high transparency, and chemical resistance [10]. On the other hand, PP’s hydrophobic character implies poor wettability and limited adhesion of proteins/cells; therefore, its surface needs to be modified to be suitable for diverse medical applications. Ejeian et al. [6] reported successful in situ crystallisation of a zeolitic imidazolate framework on the polydopamine (PDA)-modified PP membrane, which raised the surface energy, wettability, roughness, and stiffness of the substrate significantly, designing a feasible platform for multifactorial control of cell-substrate interaction.

In the presented study, polypropylene (PP) film was modified with tetraethyl orthosilicate (TEOS) under heterogeneous conditions by means of PDA/PEI chemistry (“bio-glue” layer) and a simple dip-coating procedure. Silanol groups (Si–OH) on the material´s surface can act as nucleation sites of the apatite layer, potentially forming HA in simulated body fluid (SBF) [11]. Moreover, Si-precipitating molecules can result in various nanostructured formations, depending on the reaction conditions and additives employed, thereby influencing textural features such as pore size and volume, which play a crucial role during apatite layer formation. Götz et al. [12] provided a comprehensive review of the effects of silicon on biomineralization, osteogenesis, and challenging, complex tissue formation. One part of the paper focused on mineralisation mechanisms compounds, followed by analytical techniques relevant to silicon and silicate compounds, followed by analytical techniques relevant to X-ray scattering methods. Obata et al. [13] studied the distribution state of silica derived from three different types of silicon alkoxides in a polymer matrix, i.e., TEOS, diethoxy dimethyl silane (DEODMS) and 3-amino propyl triethoxy silane (APTES), and their mineralisation ability in SBF. They concluded that composites with TEOS induce HA formation, while the composites with other silicon sources did not show this ability. Maçon et al. [14] obtained similar results for poly(3-methoxysilyl)propyl methacrylate (pTMSPMA)/SiO_2_ hybrid, which nucleated bone-like mineral on its surface after one week of immersion in SBF, whereas pure silica sol–gel glass did not. They explained this apatite-forming ability to the dipole complexation of Ca with the ester moieties of the polymer exposed after the soluble silica release from TEOS.

Dopamine was chosen in our study, since it is prone to oxidative self-polymerisation, resulting in its forming a firm PDA coating on various substrates used for convenient post-functionalisation, functionalization via non-covalent interactions, covalent coupling and surface-initiated living radical polymerization [15,16]. PDAs possess some unique features, such as bioactivity, hydrophilicity, bio-adhesion, thermal stability, the capability to functionalize with other materials, facile synthesizing, etc. [17]. Moreover, the co-deposition of dopamine with other compounds, e.g., PEI, can effectively increase modification performance [6,18].

To the best of our knowledge, there is no such strategy reporting the surface functionality effect of PDA/PEI-supported TEOS deposition on PP film’s biomineralization. Therefore, the aim of the presented research is the evaluation of the influence of the modified PP substrate´s surface on its bioactivity. Herein, based on PDA/PEI chemistry, we proposed an efficient method to functionalise the surface: Through a simple dip-coating procedure, the PP was inverted from hydrophobic to hydrophilic with the deposition of a PDA layer. Additional deposition of PEI provided amine groups for further application of TEOS, and, by its hydrolysis/condensation, self-assembly of CaP minerals on the surface of the PP film. Thus, the as-modified PP films were immersed in SBF for 3 or 7 days and characterised further in terms of morphology, physicochemical properties, topography, and mineralisation ability.

## 2. Experimental

### 2.1. Materials

**Figure 1:**
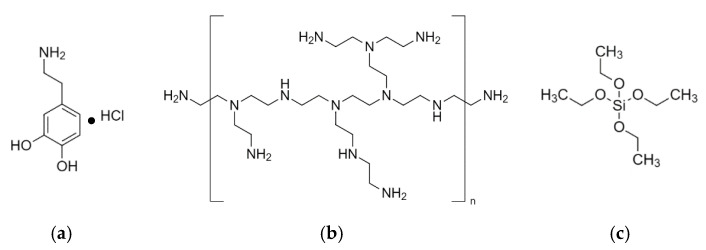
The chemical structure of: (**a**) dopa-HCl; (**b**) PEI; and (**c**) TEOS.

Polypropylene (PP) film, dopamine hydrochloride (dopa-HCl, Mw = 189.64 g/mol), polyethylene imine (PEI), tetraethyl ortho silicate (TEOS) and ethanol (EtOH, 99.8%), were purchased from Sigma-Aldrich (Taufkirchen, Germany). For the preparation of the simulated body fluid (SBF) solution, the following chemicals were purchased from Merc Co., Ltd. (Darmstadt, Germany): sodium chloride (NaCl, Mw = 58.44 g/mol), sodium hydrogen carbonate (NaHCO_3_, Mw = 84.01 g/mol), potassium chloride (KCl, Mw = 74.55 g/mol), potassium hydrogen phosphate trihydrate (K_2_HPO_4_, Mw = 228.22 g/mol), magnesium chloride hexahydrate (MgCl_2_∙6H_2_O, Mw = 203.30 g/mol), hydrochloric acid (HCl, 1.0 M, Mw = 36.46 g/mol), calcium chloride (CaCl_2_, Mw = 110.98 g/mol), sodium sulphate (Na_2_SO_4_, Mw = 142.04 g/mol) and tris(hydroxymethyl) aminomethane ((HOCH_2_)_3_CNH)_2_, Mw = 121.14 g/mol). All chemicals were utilised directly without further purification. The aqueous solutions were prepared using water purified by means of an Ultrapure Milli-Q apparatus (Millipore Corp., Billerica, MA, USA). The chemical structures of dopa-HCl, PEI and TEOS are presented in Figure 1.

### 2.2. Modification of PP Film

Before modification started, the PP films were cleaned using EtOH for 15 min and dried in a closed oven chamber at room temperature (RT).

1. Preparation of films without dopa-HCl:

A quantum of 0.1 wt.% of PEI solution was prepared in 250 mL water, with the addition of 50 mM NaCl. The PP square film (3 × 3 cm in size) was immersed for 30 min in 30 mL of pre-prepared PEI solution (PP-PEI). The as-modified films were washed with water and dried at RT. The PEI-modified PP films were additionally immersed in 30 mL of 1 wt.% TEOS solution, which was prepared in (i) 100 mL of 100% EtOH (PP-PEI-TEOS), (ii) 50 mL of EtOH and 50 mL of Milli-Q water (PP-PEI-TEOS-W), and (iii) 50 mL of EtOH and 50 mL of 0.1 M HCl (PP-PEI-TEOS-A), for 1 h at RT and 50 rpm. After that, the modified PP films were washed with EtOH and dried at RT.

2. Preparation of films with dopa-HCl:

The PP films were firstly incubated in 1% dopa-HCl solution for 24 h at RT and a rotation speed of 50 rpm (PP-PDA), and, after that, washed with water and dried at RT. In addition, the whole modification process, as well as the used solution, was repeated as described above: immersion in the PEI solution and in three different TEOS solutions. The samples were coded as follows: PP-PDA-PEI, PP-PDA-PEI-TEOS, PP-PDA-PEI-TEOS-W, PP-PDA-PEI-TEOS-A.

### 2.3. Bioactivity Assessment

The in vitro bioactivity of the modified PP films was assessed by hydroxyapatite (HA) mineralization ability in the SBF solution (pH 7.4), using a thermo-shaker SWB 25 (Thermo Haake, Karlsruhe, Germany) at a temperature of 37 ± 0.5 °C and a rotation speed of 50 rpm for 3 and 7 days. The SBF was prepared by mixing several dissolved reagents as described in [19]. The differently modified PP films (3 × 3 cm) were immersed in the SBF solution using a liquor-to-specimen weight ratio of 20:1. Thereafter, the as treated films were removed from the SBF, rinsed carefully by deionized water to eliminate soluble salts (e.g., NaCl) from the surfaces, and dried in an oven at 50 °C for 24 h. 

The baths´ pH after incubation was measured using an MA 235 pH/ion Analyzer (Mettler Toledo, Columbus, OH, USA).

### 2.4. Analytical Methods

#### 2.4.1. Fourier Transform Infrared (FTIR) Analysis

To identify molecular vibrations of the (un)modified PP surfaces, as well as surface composition and functional groups of mineral deposits, FTIR spectroscopic measurements were carried out, employing an FTIR System Spectrum GX spectrophotometer (Perkin Elmer, Waltham, MA, USA) with an Attenuated Total Reflectance (ATR) attachment. The absorbance spectra were obtained within the range of 4000–450 cm^−1^. Sixteen scans were accomplished for each sample, utilizing a resolution of 4 cm^−1^. Spectrum IR software Version 10.6.1 (Perkin Elmer, Waltham, MA, USA) was applied for analysing the data.

#### 2.4.2. Water Contact Angle (WCA) Measurement

With the aim to evaluate the hydrophilic/hydrophobic character of the (un)modified PP surfaces, the WCA measurement was accomplished using the sessile drop technique. An individual sample was placed on a horizontal table attached to a mechanical device on a Goniometer (DataPhysics Instruments GmbH, Filderstadt, Germany) including the SCA 20 program software. A micro-drop with the volume of 0.3 µL of Milli-Q water was poured onto the PP surface at an ambient temperature. The drop was illuminated by white diffuse light and observed with a tele-microscope. A clear image of the drop was transferred directly through a CCD-camera showing the drop profile. The WCA was determined from the tangent to the drop at the three-phase contact line. The average WCA values were obtained by measuring the contact angles at three various positions on the samples, and the standard deviations were calculated.

#### 2.4.3. Streaming Potential Measurement

The streaming potential measurements of the modified PP substrates were performed by means of the SurPASS 3 Electro-kinetic Analyzer (Anton Paar GmbH, Graz, Austria) using a flat plate measuring cell for solid samples as described in [20]. Briefly, a pair of an individual sample (2 × 1 cm in size) was mounted on the sample holder using double-sided adhesive tape. The gap between both samples` surfaces was adjusted to 100 ± 10 μm. As the background electrolyte, 10 mM of KCl solution was employed, whereby the analysis was started from the natural pH level to lower acidic values using 0.05 M HCl, or to higher pH values using 0.05 M KOH by means of an auto-titration unit. Three measurements were performed for individual pH, and the average streaming potential was reported.

#### 2.4.4. Atomic Force Microscopy (AFM) Analysis

The surface topography and height/area parameters (roughness) of the (un)modified PP surfaces were inspected by AFM according to the ISO 25178 standard, utilizing a Tosca 400 (Anton Paar GmbH, Graz, Austria). Herein, a tapping mode was employed, with a resonance frequency of 274 Hz at an imaging speed of 1 line/s.

#### 2.4.5. Scanning Electron Microscopy (SEM) Analysis

The surface morphology of the modified PP films was determined by SEM imaging, by means of a JSM-IT800 (Jeol, Peabody, MA, USA) ultrahigh resolution Field Emission—Scanning Electron Microscope (FE-SEM). Prior to the examination, all the samples were placed onto an adhesive carbon band fixed to a brass holder and sputtered by a thin layer of gold to create a conductive surface.

## 3. Results and Discussion

### 3.1. Modification of PP Film

**Figure 2:**
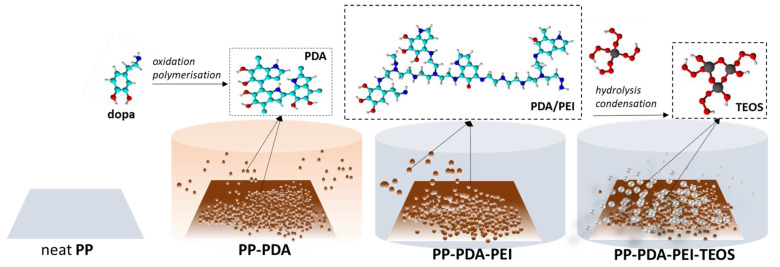
Schematic illustration of reaction mechanism for PDA/PEI-supported TEOS deposition on PP film.

**Figure 3:**
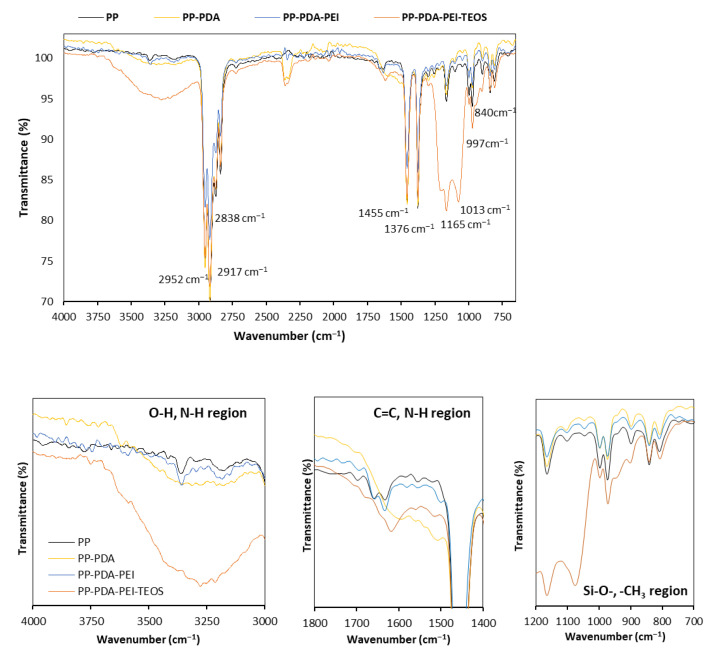
FTIR spectra of the PDA/PEI-supported TEOS deposition on the PP film (**above**) with magnification of specific regions (**below**).

The possible chemical reaction mechanism of PP films’ modification with mineralization-inducing capability included several steps (Figure 2), namely: (i) oxidation of the catechol to quinone during the self-polymerization of dopa-HCl to PDA [21], (ii) the reaction between the primary amine groups of PEI and catechol groups of dopa-HCl/PDA, which resulted in the formation of the PDA/PEI layer on the surface of the PP film via a Schiff base-type or Michael addition reaction (dark blue coloration, it can be seen in Supplementary Material), providing more positively charged functional groups [18], (iii) the hydrolysis/condensation reaction of TEOS in three different solutions, accompanied by the hydrogen bonding reaction between the PEI and TEOS [15,16], leading to the creation of a silica-modified PP-PDA/PEI film. With the aim to verify the formation of the above-mentioned layers on the surface of the PP films, FTIR transmittance spectra of the modified samples were recorded within the region of 4000–650 cm^−1^ and compared with the reference PP sample (Figure 3).

Figure 3 depicts an FTIR spectral line for both neat and modified PP foils. The spectral pattern with typical peak positions for PP include: –CH_3_ asymmetric stretching vibration at 2952 cm^−1^, –CH_2_– asymmetric stretching at 2917 cm^−1^, –CH_2_– symmetric stretching at 2838 cm^−1^, –CH_2_– symmetric bending at 1455 cm^−1^, –CH_3_ symmetric bending at 1376 cm^−1^, –CH_3_ rocking vibration at 997 and 1165 cm^−1^, and C–CH_3_ stretching vibration at 840 cm^−1^, as also interpreted fully in [22]. For better visualization, the selected regions of interest are presented separately (Figure 3—below). It is evident that the PDA modification caused a significant change in the FTIR spectrum, giving rise to a new, broad absorption band of the dopamine-modified PP sample at 3180–3500 cm^−1^ assigned to O–H and N–H stretching vibration of the PDA. A broad peak in the region 1640–1500 cm^−1^ emerged, compared to the FTIR absorption spectrum of the neat PP, where several PDA-related vibrations were present, i.e., C=C resonance vibrations in an aromatic ring, N–H bending vibrations in the catechol and amine groups of PDA [15,17,23]. The presence of PEI as an intermediate coating was not obvious in the PP-PDA-PEI, as no band at ~1573 cm^−1^, typical to bending of secondary amines (–N(R)H) in PEI, could be observed [6,24]. We speculate that the reason behind that might possibly be self-polymerization of the dopamine and PEI [25], which exclude the presence of PEI in free form. Among the new peaks revealed as the result of TEOS modification, characteristic peaks at ~1050 cm^−1^ and 800 cm^−1^ refer to the stretching and bending vibrations of the Si-O-Si bonds, respectively [26,27].

### 3.2. Bioactivity

Incubation in SBF has been used either to evaluate the mineralization ability of a material in vitro, or to produce a biomimetic coat on a material surface. In addition to biomaterials, which are intended to mineralize, the study of in vitro mineralization is also important for materials where a mineralization event is detrimental to the application, such as biomaterials used as contact lenses or vascular grafts.

#### 3.2.1. pH

In the presented study, several methods/techniques were utilized to investigate the changes on the modified PP surfaces and possible mineralization after exposure of samples to SBF media for 3 and 7 days, starting with pH measurement after the completion of the immersion process (Figure 3). The initial pH of the prepared SBF was 7.4.

**Figure 4:**
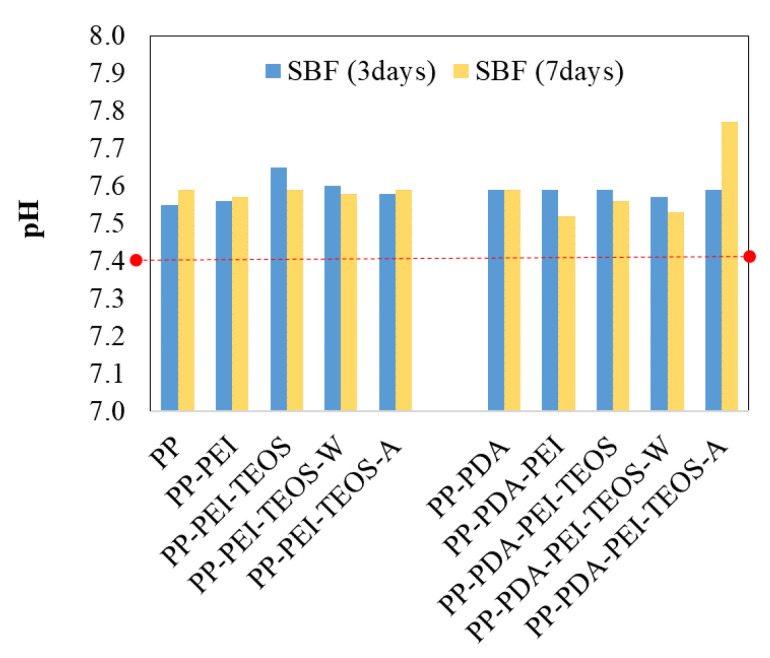
pH values of SBF medium after 3 and 7 days of films’ incubation. The initial pH of the prepared SBF was 7.4 (red dotted line).

Figure 4 shows the variations of incubation media’s pH in regard with the soaking time of the modified films (3 or 7 days). The PP film in SBF was used as a control sample. As reported by Yan et al. [28], the pH changes are associated with chemical reactions in the SBF solution during the formation of phosphate and HA for further mineralization of the modified polymer surface. The slight increase in pH could be attributed to the interchange reaction between the protons released from the substrate surface and the solution, depending on the type of functionalization compounds and the incubation time. Ghorbani et al. [17] explained that sedimentation of acidic components (e.g., HCO^3−^, HPO_4_^2−^) on the surface of PDA-modified samples can act as a stimulator of CaP nucleation, leading to the reduction of pH. On the other hand, the decrease in pH could be a consequence of the hydrolysis/dissolution of silica when TEOS was used as a modifying agent. Moreover, the actual pH of the incubation media with the TEOS-treated samples depends on the competition between the protons´ interchange reaction and hydrolysis [28].

#### 3.2.2. Surface Wettability

**Figure 5:**
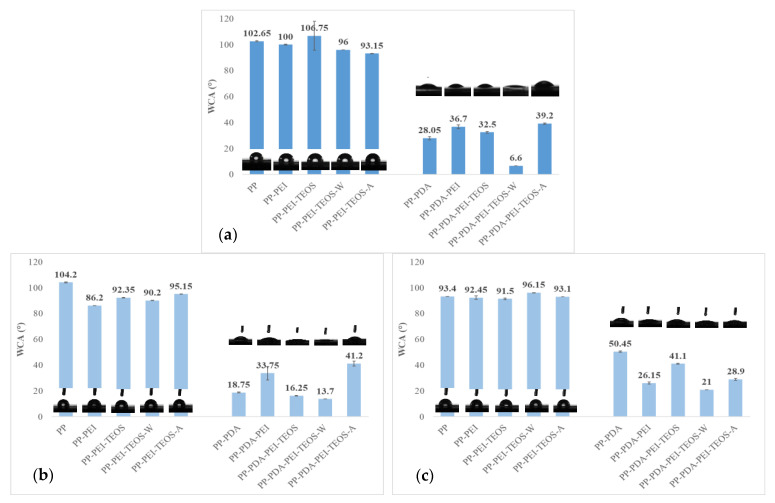
WCA of (un)modified PP films: (**a**) before incubation; (**b**) after 3 days of incubation in SBF; and (**c**) after 7 days of incubation in SBF.

One of the most important features which help to improve the cell adhesion with an implant is the surface wettability of the implant material [29]. With the aim to elucidate the role of individual compounds and their combinations on the hydrophilic/hydrophobic character of samples, the modified PP films were evaluated before and after three and seven days of incubation in SBF via the sessile drop technique using a goniometer set-up, from which the WCAs were determined, and are presented graphically in Figure 5.

From Figure 5a it can be perceived that pristine PP film (the reference) has a hydrophobic (non-polar) character with a WCA of 102.65° ± 0.35°. Modification of the PP with PEI changed the WCA negligibly. The chemical structure of PEI includes NH_2_ groups at the end of chains; the presence of these groups does not affect surface wettability. Further immersion of the PP-PEI film in TEOS changed the WCA slightly (enlarged or reduced), depending on the solvent type (100% EtOH, 50% EtOH and 50% water, or 50% EtOH and 50% HCl). On the other hand, the addition of dopa-HCl in the coating solution decreased the WCA significantly to 28.05° ± 1.2°. Similar results were reported by Ejeian et al. [6] for a PEI/PDA—modified PP membrane. This could be explained by the fact that dopamine and further PDA contain a high content of hydrophilic (−OH) groups, facilitating the reduction of interfacial tension with the water. As a result, the WCA was decreased, which leads to the superior wettability, irrespective of the presence of other compounds.

In addition, the incubation of the modified PP membranes in SBF for three (Figure 5b) or seven (Figure 5c) days changed the WCAs, which can be attributed to the existence of micro/nano structure (enhanced roughness) with compositional change of the surface, indicating in-vitro surfaces’ mineralization. Also, some changes arose within the same sample between three and seven days. In the case of PP-PDA film, WCA increased from 18.75° up to 50.45°, probably on account of the formation of cloudy-like deposits, as could be perceived from SEM results, reducing somewhat the hydrophilicity. On the contrary, the hydrophilicity increased in the case of PP-PDA-PEI-TEOS-A sample (WCA decreased from 41.20° to 28.90°) after seven days, as compared to the WCA result after three days, presumably due to the presence of Si-OH groups on the surface as explained in the Section 3.2.5.

#### 3.2.3. FTIR Spectroscopy

**Figure 6:**
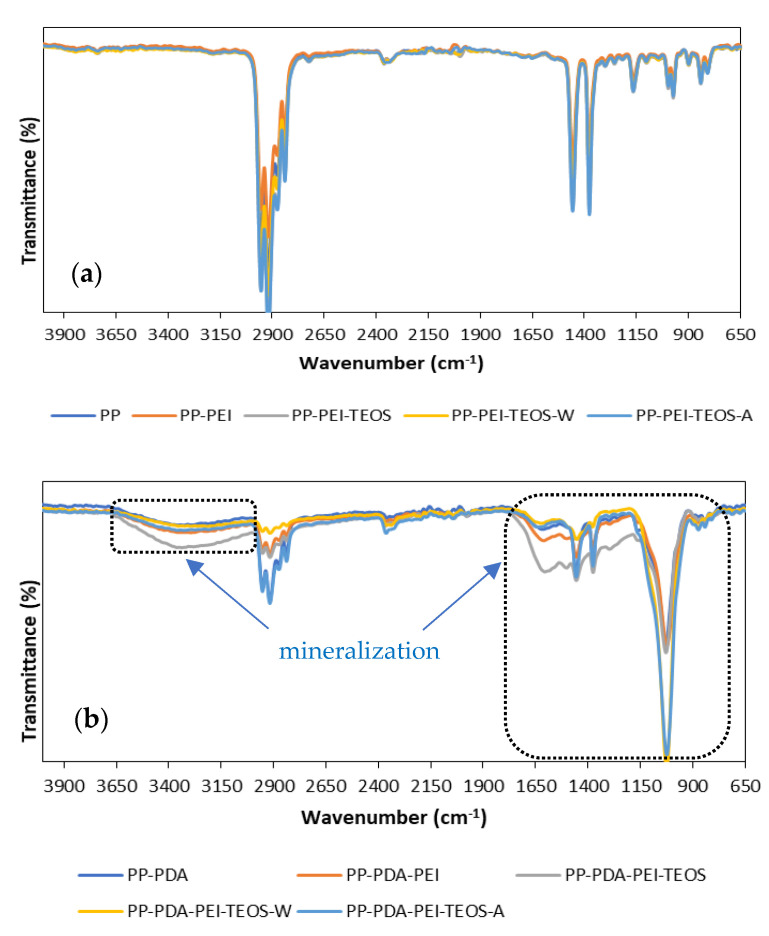
FTIR spectra of samples after 7 days of incubation in SBF: (**a**) PP films without PDA coating; and (**b**) PDA-modified PP films.

In order to examine the chemical changes on the surface of the (un)modified PP films due to incubation of the samples in an SBR medium for seven days, and, thus, the possible formation of minerals, the FTIR transmittance spectra were recorded and are presented in Figure 6.

From the curves in Figure 6a, no new band or any other significant changes of all the modified samples can be seen after 7 days of incubation in the SBF, implying mineralization disability. Contrarily, the new apatite-related peaks were formed on all the PEI/PDA-modified samples, as seen clearly from Figure 6b, suggesting the minerals` deposition, and remaining even after prolonged washing. Herein, the extremely intensive and broad peak at 1024 cm^−1^ is attributed to the phosphate asymmetrical stretching [19], as well as to the Si-O-Si symmetrical stretching of the hydrolysed TEOS [26,30], at 1296 cm^−1^ to the P-OH bending, at 1455 cm^−1^ to the carbonyl groups, and the broad band at ~3500 cm^−1^ corresponded to the stretching vibrations of the −OH groups [19]. The small peak at ~875 cm^−1^ is related to the C-O vibrations of hydroxyapatite and the intensified peak at ~1610 cm^−1^ to O-H stretching [17]. Moreover, the PP-related peaks at 2952, 2917, 2838, 1455 and 1376 cm^−1^ are less intensive, presumably on account of the enlarged thickness of the apatite layer.

Since the FTIR results of the PDA-excluded samples (Figure 6a) did not show any significant changes after incubation in SBR, only the PDA/PEI-modified samples after seven days of SBR exposure were further selected for zeta potential measurement, as well as for surface morphology and topography inspection.

#### 3.2.4. Zeta Potential

**Figure 7:**
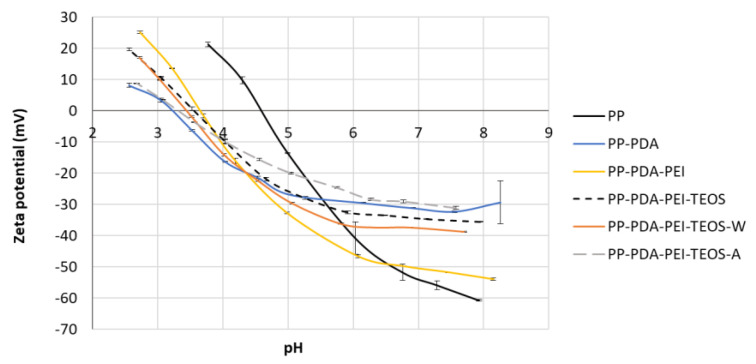
Zeta potential of (un)modified PP films after 7 days of incubation in SBF at different pH.

The zeta potential of materials is of great scientific and technological importance in many fields, indicating their surface electric features, and, therefore, it is connected strongly with the development of biochemical and physicochemical processes at the substrates´ surfaces [31,32]. The zeta potential of the modified PP films after 7 days of incubation in SBF was determined by streaming potential measurements at different pH and are presented in Figure 7.

An almost linear decrease of the titration curve can be observed from Figure 7 for the pure PP film when increasing the pH, which is consistent with the results obtained by [33]. The lower values of the zeta-potential at individual pH indicate a negative charge of the material surface [32]. The modified PP films manifested a similar decrease, but with the lower absolute values of zeta-potential between pH 6 to 8, revealing specific interactions between different compounds, as well as the possible self-assembly of CaP, and, thus, supporting the results obtained by FTIR and WCA. The dielectric constant of different solvents (EtOH, EtOH/H_2_O or EtOH/HCl) in the case of PDA/PEI-supported TEOS deposition could have some impact on the charge density of the samples, as explained in [17]. In addition, the Isoelectric Point (IEP) that indicates an amphoteric behaviour of the film was found to be at a lower pH (~3.5) after surface modification/incubation in SBF as compared to the pure PP (IEP at pH 4.6).

#### 3.2.5. Surface Morphology and Topography

**Figure 8:**
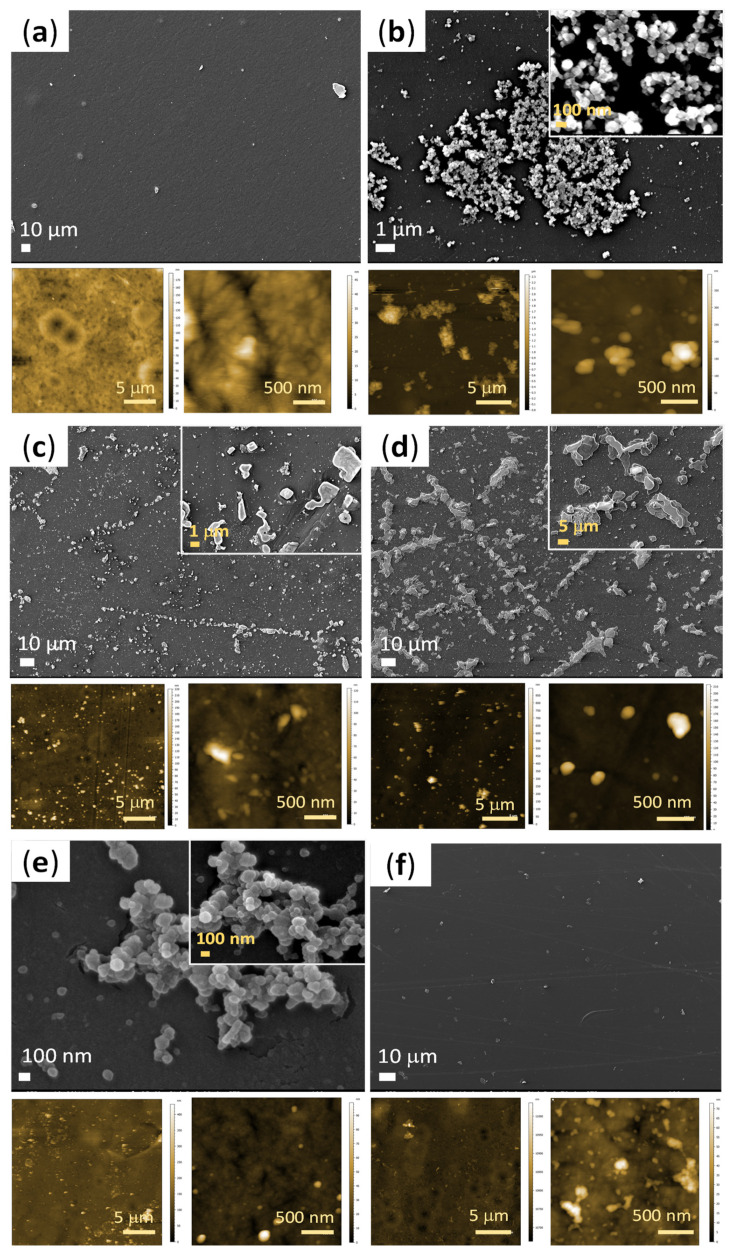
FE-SEM images of samples after 7 days of incubation in SBF: (**a**) PP; (**b**) PP-PDA; (**c**) PP-PDA-PEI; (**d**) PP-PDA-PEI-TEOS; (**e**) PP-PDA-PEI-TEOS-W; (**f**) PP-PDA-PEI-TEOS-A; and corresponding AFM 2D-height topography maps at different magnifications.

The surface characteristics of a material may influence not only the cell recruitment, but also the biofilm accumulation. Thus, the morphological and topographical (surface roughness) characteristics of the modified PP structures were inspected after 7 days of incubation in SBF, and the results are presented in Figure 8 and Table 1.

As can be perceived from the SEM images in Figure 8f, the PP-PDA-PEI-TEOS-A sample had smooth surface morphology with some impurities, similar to the uncoated PP film (Figure 8a), implying unsuccessful deposition of CaP minerals. Thus, the intensive peak for the PP-PDA-PEI-TEOS-A sample at 1024 cm^−1^ notable on Figure 6a, is attributed to the Si-OH groups of TEOS and not phosphate-stretching. As explained by Rubio et al. [26] the HCl catalyst in the TEOS solution accelerated the 3D crosslinking of the Si-OH groups through polycondensation reaction, as compared to the slower hydrolysis/polycondensation when TEOS is diluted in EtOH or in combination of EtOH and water. Thus, fewer free silanol groups were available for the reaction with the Ca^2+^ ions in the SBF. Consecutively, the sample had the lowest roughness in comparison to the other modified samples, as seen from both Table 1 and the 2D color-coded height images of AFM measurement (two different magnifications), where the dark pixels designate the low points (valleys) and light pixels the high points (peaks). On the contrary, deposits with a cloudy-like shape were found on both the PP-PDA (Figure 8b) and PP-PDA-PEI-TEOS-W (Figure 8e) surfaces, indicating successful mineralization based on interactions between the abundant catecholamine moieties in the PDA (or silanol groups in TEOS) and available Ca^2+^ ions in the SBF [13,17].

Less equal and more agglomerated deposits on the PP-PDA had an effect on the higher surface roughness with an arithmetic mean height of 135 nm and areal height difference of 376 nm as compared to PP-PDA-PEI-TEOS-W (14.1 and 31.4 nm, respectively). In addition, a lot of relatively large undefined forms were noted on the surface of the PP-PDA-PEI-TEOS (Figure 8d), influencing the surface roughness. Presumably, those formations were attributed to the silica coatings. Szewczyk et al. [34] mentioned that the self-formed hydroxyapatite in vitro ranges typically from 30 to 60 days to cover the mesoporous silica fully. Since the roughness affects the anchoring and attachment property [35], the CaP deposition could eventually start in our case if the immersion of the sample in SBF were to continue.

## 4. Conclusions

In the presented study, the modification of PP film was realised successfully by TEOS under heterogeneous conditions via PDA/PEI chemistry using a facile dip-coating procedure, as confirmed by the FTIR results. The deposition of dopamine hydrochloride, which oxidised into PDA during self-polymerisation, changed the hydrophobic character of the PP surface into hydrophilic (the WCA decreased from 103° for the pure PP film down to 28° for the PDA-modified film), and, thus, it became more bioactive with a higher possibility for the self-assembly of CaP minerals after exposure in the ion-rich SBF solution, in comparison to the PEI-modified samples. In addition, the PEI in the PDA/PEI-functionalised samples provided more positively charged amine groups for the hydrogen bonding between the PEI and hydrolysed TEOS, enabling the efficient interaction between the PP-modified films and mineral ions (e.g., Ca^2+^), which facilitated the deposition of minerals from the ion-saturated SBF solution. Moreover, the roughness was enhanced as compared to pure PP, which affects the attachment capability and self-assembly of minerals. Thus, the morphological study reveals successful deposition of the formation of CaP with a cloudy-like shape on the PDA/PEI/TEOS-modified surface when the TEOS was diluted in the combination of ethanol and water. Considering that this simple approach is facile and robust enough to allow further specific functionalisation for adjusting surface properties, these findings may lead to the development of new efficient strategies for surface functionalisation of inert PPs that are of great potential to the biomedical field.

## Figures and Tables

**Table 1:** AFM-based surface roughness parameters of samples after 7 days of incubation in SBF.

Sample	Root-Mean-Square Height (nm)	Skewness	Kurtosis	Arithmetic Mean Height (nm)	Areal Height Difference (nm)
PP	13.4	0.0124	5.62	9.79	24.5
PP-PDA	207	2.81	11.8	135	376
PP-PDA-PEI	19.2	4.20	24.5	9.99	19.2
PP-PDA-PEI-TEOS	59.0	6.01	48.3	25.2	36.5
PP-PDA-PEI-TEOS-W	26.5	4.70	35.7	14.1	31.4
PP-PDA-PEI-TEOS-A	19.6	3.36	22.8	12.0	29.0

## Data Availability

The data presented in this study are available on request from the corresponding author. The data are not publicly available due to privacy.

## Supplementary Materials

The following supporting information can be downloaded at: https://www.mdpi.com/article/10.3390/polym15040902/s1.
