# Supplementary figures and images for: Dopamine-Assisted Modification of Polypropylene Film to Attain Hydrophilic Mineral-Rich Surfaces
Source: Polymers (Basel). 2023 Feb 11;15(4):902. doi: 10.3390/polym15040902 (PMC9962719; doi:10.3390/polym15040902)

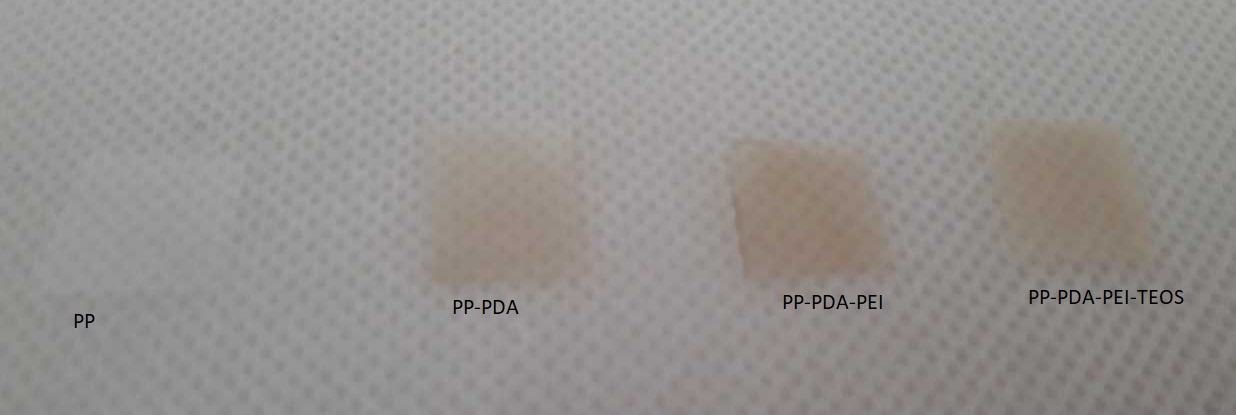

Supplement: Supplementary file 1 [file polymers-15-00902-s001.zip › polymers-2203374-supplementary.jpg]
